# Polarimetric-Phase-Enhanced Intensity Interrogation Scheme for Surface Wave Optical Sensors with Low Optical Loss

**DOI:** 10.3390/s18103262

**Published:** 2018-09-28

**Authors:** Yuhang Wan, Zheng Zheng, Mengxuan Cheng, Weijing Kong, Kai Liu

**Affiliations:** 1 School of Electronic and Information Engineering, Beihang University, 37 Xueyuan Road, Beijing 100083, China; yuhangwan@buaa.edu.cn (Y.W.); zhengzheng@buaa.edu.cn (Z.Z.); cmx_325@163.com (M.C.); 2 Collaborative Innovation Center of Geospatial Technology, 129 Luoyu Road, Wuhan 430079, China; 3 Beijing Advanced Innovation Center for Big Date-based Precision Medicine, Beihang University, 37 Xueyuan Road, Beijing 100083, China; 4 School of Electronic Engineering, Tianjin University of Technology and Education, 1310 Dagu South Road, Tianjin 300222, China; 40110@tute.edu.cn

**Keywords:** optical instruments, surface waves, optics at surfaces, refractive index sensors

## Abstract

A polarimetric-phase-enhanced intensity interrogation scheme leveraging the polarization-dependent sharp phase change induced by the surface wave excitation at a low-optical-loss sensor’s surface is proposed and experimentally demonstrated. Based on a simple setup with no moving parts during interrogation, a polarimetric-phase-enhanced intensity can be obtained by subtracting the reflected intensities of two beam polarization states. Our results show a ~4-fold sensitivity increase compared to traditional intensity detection schemes for similar sensors. As novel surface wave optical sensors are designed and engineered with optimized phase responses, this scheme offers a low-complexity solution for such devices instead of traditional phase interrogation schemes.

## 1. Introduction

Optical sensing technologies have become essential for many applications of chemical and biological analytes measurements. Among them, the demand for high-sensitivity, label-free, and real-time diagnostic tools has motivated extensive research in the field of surface-wave-based optical sensing techniques. Surface plasmon resonance (SPR) based on the surface plasmon wave (SPW) propagating along the interface between a dielectric and a thin metal film such as gold or silver has been widely studied for the past thirty years and has been regarded as the gold standard for biomolecular interaction analysis [1,2,3]. As better performance is on demand for wider applications, such as higher sensitivity for lower concentration or smaller molecular detection, novel optical surface wave sensors and their associated sensing schemes have been proposed. For example, the sensing scheme based on Bloch surface wave (BSW) excited in a truncated one-dimensional photonic crystal structure has been demonstrated to be an effective alternative to SPR with unique characteristics, due to its much-reduced propagation loss at the surface [4,5,6,7,8]. In contrast to the SPR sensors based on lossy metallic surfaces, such dielectric devices can have much-reduced optical propagation loss for the optical surface wave. This further results in much sharper angular and phase response curves but significant reduced reflectivity contrast. Therefore, it would be interesting to explore optical interrogation schemes that could better fit these new types of optical surface wave sensors.

Conventional interrogation methods for surface-wave-based sensors could be categorized in two dimensions: by the scanning parameter, such as the incident angle or the incident wavelength; and by the measured parameter, the intensity, or the phase of the reflected light. The intensity detection is easy to implement. Therefore, it is applied in most of the commercial instruments. Among the configurations based on the intensity measurement, the real-time intensity monitoring with fixed incident angle and wavelength has been attractive for its simplicity and capability for high-throughput sensing scheme. Despite the benefits, the main disadvantage of this scheme is that the sensitivity might be not as high as the other configurations. The sensitivity of the intensity detection method depends on the sharpness of the resonance dip. As is known, the profile of the surface wave resonance dip strongly depends on the optical loss of the sensor. For metal induced sensors such as SPR devices, the reflectivity could approach zero with a wider shape. For metal-free devices such as BSW sensors, due to the reduction of the optical loss, the resonance dip would be much narrower, as the full width at half maximum (FWHM) for BSW resonance could be one tenth of that for the SPR, resulting in an improved sensitivity for the intensity detection [9]. However, the lower the optical loss, the shallower the reflectivity, which leads to a worse signal-to-noise ratio and increases the difficulty of the intensity measurement.

On the other hand, the phase detection has the potential to achieve higher sensitivity as abrupt phase jump occurs with the excitation of the surface wave. Lots of effort have been carried out for phase detection of the SPR during past decade [10,11,12]. However, the phase is difficult to measure directly, but needs to be retrieved via interferometric schemes in either spatial, temporal, or spectral domain, where the system setups are usually bulky and complex compared to intensity interrogation setups. For BSW sensors, due to the absence of the metal, the phase change of the low-optical-loss device would be much more significant than the SPR [13,14], which makes the application of phase-sensitive sensing schemes even more attractive. Recently, a few reports on phase-sensitive BSW sensing techniques have been proposed, but as in the conventional SPR configurations, the scheme is either based on rotating the polarizer and the analyzer in an ellipsometric configuration [15], or on controlling the phase retarder [16] to compare the difference between the incident and the reflected beam.

In this paper, a novel polarimetric-phase-enhanced intensity detection scheme is proposed for the low-optical-loss surface wave sensors, in which no moving part is required during the sensing experiment. In a setup similar to the traditional intensity interrogation ones, a simple and compact in-line optical configuration is designed where the phase change induced by the sensor is associated with the intensity term that could be obtained by subtracting the measured reflected intensities of two different polarized light through two channels simultaneously. A BSW sensor with proper design of the truncated one-dimensional photonic crystal structure where the surface wave could be excited for aqueous external medium is used for experimental demonstration. The polarimetric-phase-enhanced intensity signals are then captured for different sample solutions, which shows improved sensitivity performance compared with current intensity detection schemes.

## 2. Proposed Scheme and Experimental Setup

**Figure 1:**
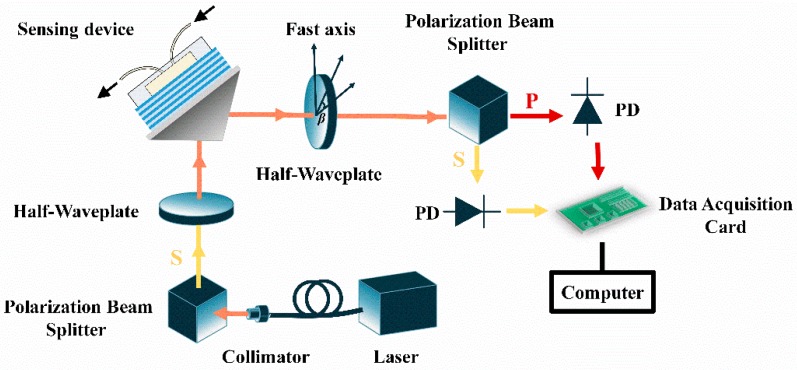
Schematic diagram of the proposed scheme.

The schematic diagram of a typical proposed polarimetric-phase-enhanced intensity detection setup is shown in Figure 1, where the surface wave sensor is excited based on the conventional Kretschmann-Reather prism-coupling configuration. In our system, a fiber-coupled laser emitting at 980 nm is used as the light source, where the output is collimated from the fiber endface and then followed by a polarization beam splitter and a half wave plate with the azimuth angle setting at β_0_ = 22.5°. These two components jointly function as a 45° polarizer to set the intensity of the P polarization and S polarization components in the beam illuminating the sensor to be equal. Then the light is coupled through the side facet of an equilateral-triangle coupling prism to the sensor chip. As described later, the BSW in the sensor could be excited for P polarization with a proper incident angle, when the surface of the device is exposed to water. A flow-cell made of Polydimethylsiloxane (PDMS) is attached to the terminal layer of the BSW sensor device to introduce aqueous samples to the surface. The prism-coupling apparatus is controlled by a motorized rotation stage to alter the incident angle for the excitation of the sensor. Then the reflected light passes through another half wave plate with an azimuth angle β, which is followed by another polarization beam splitter to separate the P polarization and S polarization components. Both polarization components are directly measured by two independent photodiode detectors (Newport, Irvine, CA, USA) respectively. Their outputs are recorded by a data acquisition card through two different channels simultaneously.

For the proposed experimental setup, the Jones matrix vector of the 45° polarized light after the polarization beam splitter and the half wave plate is given by:

(1) $$P=\frac{1}{\sqrt{2}}\left[\begin{array}{c} 1\\ 1 \end{array}\right]\;$$

The Jones matrix of the BSW sensor could be expressed as:

(2) $$\;\mathrm{BSW}=\left[\begin{array}{cc} re^{i\varphi } & 0\\ 0 & 1 \end{array}\right]\;$$

where *r* and *φ* are the field reflectivity and phase shift introduced by the BSW excitation, respectively, both of which could be calculated through the Fresnel equation. The Jones matrix of the second half wave plate with an azimuth angle *β* is:

(3) $$\;H=\left[\begin{array}{cc} \cos2\beta & \sin2\beta \\ -\sin2\beta & \cos2\beta \end{array}\right]\;$$

For each polarization output, the second polarization beam splitter can be expressed as:

(4) $$PBS_{p}=\left[\begin{array}{cc} 1 & 0\\ 0 & 0 \end{array}\right],\;PBS_{s}=\left[\begin{array}{cc} 0 & 0\\ 0 & 1 \end{array}\right]\;$$

Therefore, the electrical field of the output for the *P* and *S* polarization can be expressed respectively, as

(5) $$\;E_{p}=\frac{1}{\sqrt{2}}\left[\begin{array}{cc} 1 & 0\\ 0 & 0 \end{array}\right]\left[\begin{array}{cc} \cos2\beta & \sin2\beta \\ -\sin2\beta & \cos2\beta \end{array}\right]\left[\begin{array}{cc} re^{i\varphi } & 0\\ 0 & 1 \end{array}\right]\left[\begin{array}{c} 1\\ 1 \end{array}\right]\;$$

(6) $$\;E_{s}=\frac{1}{\sqrt{2}}\left[\begin{array}{cc} 0 & 0\\ 0 & 1 \end{array}\right]\left[\begin{array}{cc} \cos2\beta & \sin2\beta \\ -\sin2\beta & \cos2\beta \end{array}\right]\left[\begin{array}{cc} re^{i\varphi } & 0\\ 0 & 1 \end{array}\right]\left[\begin{array}{c} 1\\ 1 \end{array}\right]\;$$

The output intensities of both polarization components are given by:

(7) $$I_{p}=\left|E_{p}{}^{2}\right|=\frac{1}{2}{(r}^{2}\cos^{2}2\beta +\sin^{2}2\beta +r\times \sin4\beta \times \cos\varphi )\;$$

(8) $$I_{s}=\left|E_{s}{}^{2}\right|=\frac{1}{2}{(r}^{2}\sin^{2}2\beta +\cos^{2}2\beta -r\times \sin4\beta \times \cos\varphi )\;$$

Based on the above equations, when the azimuth angle of the second half wave plate *β* is set at 22.5°, the measured intensities for the *P* and *S* polarization component can be expressed as:

(9) $$I_{p}=\frac{1}{4}{(r}^{2}+\;1)+\frac{1}{2}r\times \cos\varphi \;$$

(10) $$I_{s}=\frac{1}{4}{(r}^{2}+\;1)-\frac{1}{2}r\times \cos\varphi \;$$

Therefore, a phase-sensitive intensity item *I_PA_* could be obtained by subtracting the measured two signals related to two different polarization components:

*I_PA_* = I*_p_* − I*_s_* = *r*cos*φ*

**Figure 2:**
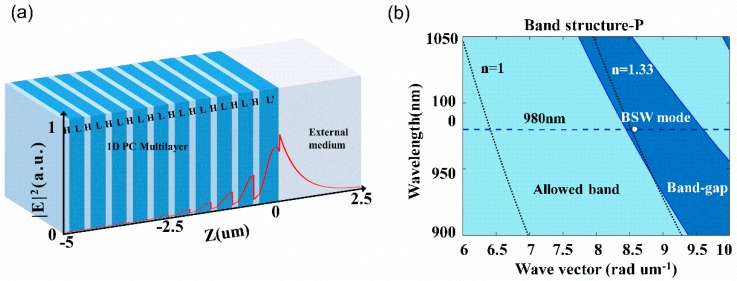
(**a**) Schematic of the BSW sensor and the normalized electric field distribution of the excited BSW; (**b**) Simulated band structure of the 1D PC structure for P polarization and the BSW mode excited. The light blue region is the allowed band and the dark blue region is the band-gap. Dotted lines are light lines for air (*n* = 1) and water (*n* = 1.33) respectively.

The sensor device used to excite the BSW mode in this study is sketched in Figure 2a, which is based on a truncated 1D PC multilayer structure and can be expressed as substrate /(HL)^9^HL’/cladding. For P-polarized incident light at the wavelength of 980 nm, the device is designed to be able to excite the BSW mode with water as the cladding (*n* = 1.33). The substrate is made up of ZF10 glass (*n_S_* = 1.668), with TiO_2_ and SiO_2_ evaporated alternatively as high index layer (H, *n_H_* = 2.3) and low index layer (L, *n_L_* = 1.434) respectively, where the terminal layer adjacent to the external medium is also SiO_2_ (L’) with a different thickness. In the 1D PC structure, the thicknesses of the high index layer, the low index layer and the terminal layer are 163 nm, 391 nm, and 500 nm, respectively. To represent the intrinsic loss of the material and the scattering loss of the surface induced in the fabrication process, an extinction coefficient is added to the imaginary part for the refractive index of the TiO_2_ layers for calculation to match the experimentally observation as 2 × 10^−4^.

**Figure 3:**
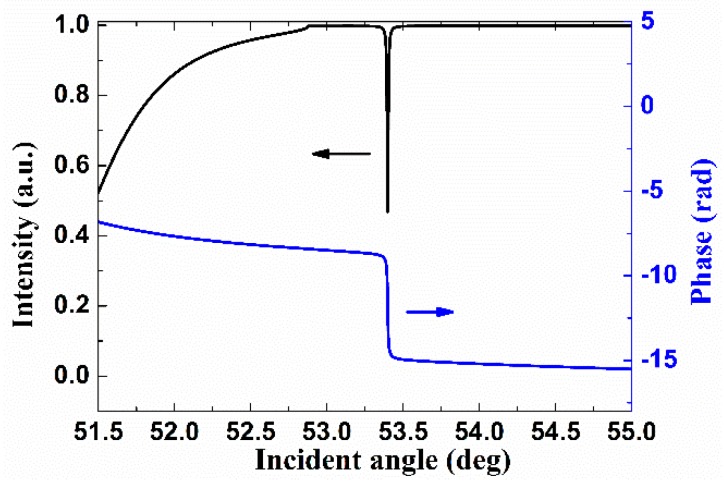
Simulated angular reflectance and phase response when the external medium is water for P-polarized input.

The excited BSW mode is marked in the band structure of the 1D PC structure as shown in Figure 2b, where the band structure is calculated for the P-polarized input [17]. For the considered wavelength, it can be noted that the rising edge of the forbidden band is just to the left of the light line for water (*n* = 1.33). By calculating the effective refractive index of the excited BSW mode (*n*_eff_ = *n*_s_sinθ = 53.4°), it is noted that the marked BSW mode (red solid circle) just sits in the forbidden band and lies slightly beyond the light line for water, suitable for sensing applications in an aqueous solution environment. The excited BSW mode could also be observed in the simulated angular reflectance using Fresnel equations as shown in Figure 3, represented by the sharp attenuation dip beyond the total internal reflection, where the phase response is plotted for comparison as well, showing an abrupt phase jump correspondingly.

The normalized electric field distribution for the excited BSW is shown in Figure 2a. It shows that the electric field is highly concentrated and enhanced at the interface with a decaying tail into the neighboring medium above the surface. It is also noted that the penetration depth of the BSW into water (at 1/e) is at the order of ~1 μm, which is much greater than that of the SPW. Therefore, the resonance will be more sensitive to the change of the bulk refractive index above the surface.

## 3. Results and Discussions

**Figure 4:**
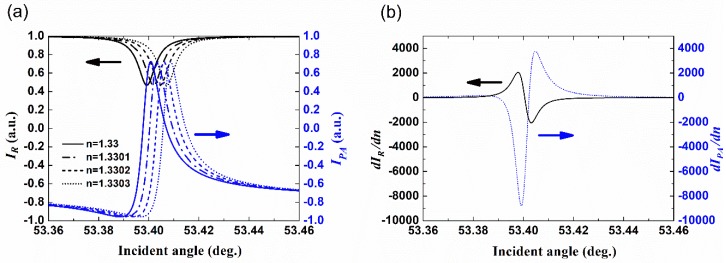
(**a**) The polarimetric-phase-enhanced intensity and the reflectance for different refractive indices with the BSW excitation. Solid lines: *n* = 1.33; (**b**) The sensitivities of the polarimetric-phase-enhanced intensity and the reflectance.

In Figure 4a, the simulated polarimetric-phase-enhanced intensity *I_PA_* is plotted together with the reflected intensity *I_R_* =|*r*|^2^ based on the above assumption and device, with the refractive index of the external medium changing from 1.33 with a step of 0.0001. It shows that the subtracted intensity changes more abruptly in amplitude with either the incident angle or the refractive index of the external medium, compared to the reflected intensity for one single polarization. To further confirm that, Figure 4b plots the first derivative to the refractive index of both the polarimetric-phase-enhanced intensity (*dI_PA_*/*dn*) and the reflectance (*dI_R_*/*dn*), showing a ~4-fold increase of the maximum sensitivity. It would be easy to conclude through the calculation that with a lower optical loss, the sensitivity enhancement of *I_PA_* would be more significant.

**Figure 5:**
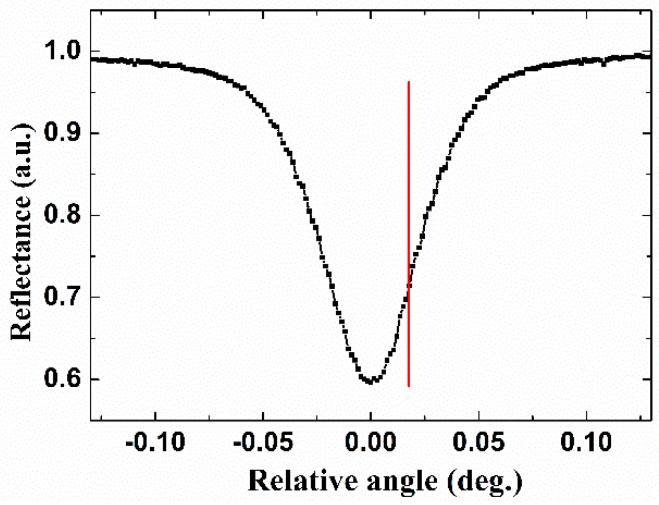
The measured reflected intensity with the BSW excitation for water cladding. (red solid line: the working angle).

The excitation of the BSW mode could be observed through the angular scanning either for the reflected intensity [9], or the phase-related Goos-Hanchen effect [14] as our previous studies shown, at the beginning of the experiment. To experimentally characterize the basic characteristics of the sensor before making any measurement, the incident light is initially set as P polarization by setting the azimuth angle of the first half wave plate to 0°, as the BSW sensor device is designed for P polarization with water cladding. A photodiode is placed directly after the BSW sensor device to measure the reflected intensity to locate the angular position where the BSW is excited. The measured reflected intensity near the BSW excitation is shown in Figure 5. After normalization, the minimum of the reflectance drops to ~0.6, which agrees with the simulation in Figure 4a. We note that the finite width of beam illumination would lead to shrinkage and broadening of the reflectance curve, and the beam width effect could be eliminated by performing deconvolution on the experimental data in further for more accurate comparison with the simulation [18]. The angular position of the maximal sensitivity of *I_PA_* is close to that of *I_R_*, which lies around the angular position 1/4 of the full height from the dip of the angular reflectivity curve, as implied in Figure 4. Therefore, the position of the working angle is set according to the simulation, as shown in Figure 5.

**Figure 6:**
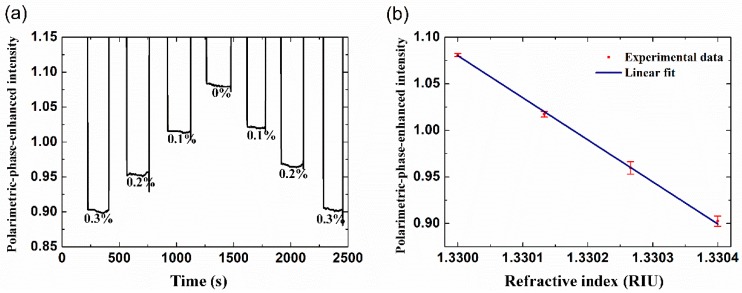
(**a**) Real-time record of the subtracted, phase-induced intensity *I_PA_* with different solutions; (**b**) the linear fit between the measured *I_PA_* and the refractive index of the solution.

During the sensing experiment, as discussed above, the working angle is fixed around the position where the BSW is excited and the polarimetric-phase-enhanced intensity is most sensitive to the external medium refractive index change. Different glycerol solutions in de-ionized (DI)-water with the concentrations 0.1–0.3 wt.% (the equiv. index difference =1.17 × 10^−4^ RIU [19]) are injected into the flow-cell as the test samples. Figure 6a shows the real-time record of the polarimetric-phase-enhanced intensity for different solutions varying from pure water to the highest concentrated glycerol solution with a concentration change of 0.1% for each step. During the monitoring, air bubbles are injected before and after each solution for isolation, to avoid the mixture of different samples. It can be found that the repeatability as well as the stability of the system is quite well. The relation between the measured *I_PA_* and the refractive index is plotted in Figure 6b, which shows good linearity. By a linear fit of the experimental data, a slope representing the inverse of the system sensitivity of 2.0 × 10^−3^ RIU /V is obtained, compared to that measured with the same sensor device in an intensity detection configuration of 8.4 × 10^−3^ RIU/V as reported earlier [9]. A ~4-fold increase of sensitivity is obtained through the experiment, which agrees well with the simulation estimation. As is known that the loss of the dielectric stack influences the phase change as well as the reflectance of the BSW significantly [13], the sensitivity could be further improved, by properly designing the structure stack and the optical loss.

## 4. Conclusions

A polarimetric-phase-enhanced intensity detection scheme is proposed and experimentally demonstrated for a surface wave sensor with low optical loss. By measuring an all dielectric multilayer structure designed to excite the BSW for P polarization with a sharp phase change as the low-optical-loss sensor, our experimental results show that the polarimetric-phase-enhanced intensity signal of the low loss BSW sensor is very sensitive to the refractive index change of the surface. Improvement in sensitivity compared to the traditional intensity detection scheme with the same sensor structure is demonstrated. Because of the relatively simple system setup compared to the phase measurement setups, this scheme could be an interesting alternative to leverage the phase-response-oriented sensors with the benefit of traditional intensity interrogation systems. It also has the potential for high-throughput label-free sensing applications, which so far is more difficult to realize for phase interrogation schemes.
